# Patient-tailored risk assessment of obstructive coronary artery disease using Rubidium-82 PET-based myocardial flow quantification with visual interpretation

**DOI:** 10.1007/s12350-023-03237-z

**Published:** 2023-04-19

**Authors:** S. S. Koenders, J. A. van Dalen, P. L. Jager, M. Mouden, C. H. Slump, J. D. van Dijk

**Affiliations:** 1 Department of Nuclear Medicine, Isala Hospital, PO Box 10400, 8000 GK Zwolle, The Netherlands; 2 Department of Medical Physics, Isala Hospital, Zwolle, The Netherlands; 3 Department of Cardiology, Isala Hospital, Zwolle, The Netherlands; 4 Technical Medical Centre, University of Twente, Enschede, The Netherlands

**Keywords:** Myocardial blood flow, PET myocardial perfusion imaging, ^82^Rb, MBF, Segmental MFR

## Abstract

**Introduction:**

Our aim was to estimate the probability of obstructive CAD (oCAD) for an individual patient as a function of the myocardial flow reserve (MFR) measured with Rubidium-82 (Rb-82) PET in patients with a visually normal or abnormal scan.

**Materials and Methods:**

We included 1519 consecutive patients without a prior history of CAD referred for rest-stress Rb-82 PET/CT. All images were visually assessed by two experts and classified as normal or abnormal. We estimated the probability of oCAD for visually normal scans and scans with small (5%–10%) or larger defects (> 10%) as function of MFR. The primary endpoint was oCAD on invasive coronary angiography, when available.

**Results:**

1259 scans were classified as normal, 136 with a small defect and 136 with a larger defect. For the normal scans, the probability of oCAD increased exponentially from 1% to 10% when segmental MFR decreased from 2.1 to 1.3. For scans with small defects, the probability increased from 13% to 40% and for larger defects from 45% to > 70% when segmental MFR decreased from 2.1 to 0.7.

**Conclusion:**

Patients with > 10% risk of oCAD can be distinguished from patients with < 10% risk based on visual PET interpretation only. However, there is a strong dependence of MFR on patient’s individual risk of oCAD. Hence, combining both visual interpretation and MFR results in a better individual risk assessment which may impact treatment strategy.

**Supplementary Information:**

The online version contains supplementary material available at 10.1007/s12350-023-03237-z.

## Introduction

The use of myocardial blood flow (MBF) quantification using Rubidium-82 (Rb-82) in myocardial perfusion imaging (MPI) with positron emission tomography (PET) is rapidly increasing.^1–3^ This is mainly caused by the availability of Strontium-82/Rb-82 generators and the better accuracy of PET in comparison to SPECT imaging.^4,5^ Global myocardial flow reserve (MFR) values provide incremental prognostic value over visual interpretation of the PET scans and help better identify patients at risk of cardiac events.^6,7^ To prevent the development of cardiac events, a patient-tailored risk assessment of obstructive CAD (oCAD) is essential for choosing an appropriate treatment strategy. PET-based MFR in combination with visual assessment can be used for this purpose, as in clinical practice, PET is used to assess the presence, extent, and functional importance of oCAD.^7,8^ However, in assessing patient’s risk of oCAD, it is unclear how MFR should be combined with visual assessment, especially when they are discordant. How should the readers interpret patients with a normal scan and low MFR, or patients with an abnormal scan but high MFR? Hence, our aim was to estimate the probability of oCAD for an individual patient as a function of the MFR in patients with a visually normal scan as well as in patients with a visually abnormal scan.

## Materials and methods

### Study population

We retrospectively included 1519 patients referred for rest and regadenoson-induced stress Rb-82 PET/CT (GE Discovery 690, GE Healthcare) without a prior history of CAD and of whom at least one-year follow-up was available. As this study was retrospective, approval by the medical ethics committee was, therefore, not required according to Dutch law. Nevertheless, all patients provided written informed consent for the use of their data for research purposes.

### Patient preparation, data acquisition, and reconstruction

All subjects were asked to refrain from caffeine containing substances for 24 h and to discontinue dipyridamole containing medication for 48 h prior to imaging. All patients underwent a rest scan followed by a regadenoson-induced stress scan. The PET/CT acquisition and reconstruction protocol have been described previously.^9^ In short, we acquired a low-dose CT scan prior to MPI during free breathing to provide an attenuation map of the chest. PET list-mode data were acquired in rest during 7 min directly after administration of 740 MBq Rb-82. Ten minutes after the first activity bolus, we induced pharmacological stress by administrating 400 μg (5 mL) of regadenoson over 10 s. Subsequently, a second dose of 740 MBq Rb-82 was administered, followed by another PET acquisition. Attenuation correction was applied to all data on the PET system after semi-automatic registration of CT and PET data. We reconstructed the dynamic PET datasets using 26 time frames (12 × 5 s, 6 × 10 s, 4 × 20 s and 4 × 40 s). Static rest and stress images were reconstructed from PET data acquired between 2:30 and 7:00 min after Rb-82 administration.

### Data analysis

We used Corridor4DM (v2016.02.64) software to post-process the dynamic images.^10^ All static Rb-82 PET images were visually assessed by two expert readers and classified as normal or as abnormal, where abnormal was defined as images showing a reversible and/or irreversible perfusion defect. In addition, perfusion defects were either rated as small (5%–10% of the left ventricle) or larger (> 10% of the left ventricle).^11^ The one-tissue compartment model of Lortie et al^12^ was used to calculate the MBF from the time activity curves (TACs) of the image-derived left ventricle blood pool and the myocardium. The dynamic images were visually inspected for the presence of myocardial creep and manually corrected if necessary.^9^ MFR was calculated as the ratio of stress MBF to rest MBF. In our previous study, we showed the added diagnostic value for regional MFR over global MFR.^13^ In addition to the global MFR (globMFR), we therefore also determined MFR in each of the 17 left ventricular myocardial segments.^13^ We defined regional MFR as the lowest flow reserve in all 17 segments (segMFR).

Next, we estimated the patient’s probability of having oCAD for visually normal and abnormal scans and for visual vs. small and larger defects as a function of both globMFR and segmMFR. To obtain proper statistics for calculating the probability of oCAD, we divided patients into quintiles based on globMFR and segmMFR for the patient group with visually normal scans and for either the patient group with visually abnormal scans or patient groups with small or larger defects. For each quintile, we calculated the mean globMFR and mean segmMFR, and corresponding standard error. Next, we fitted the mean MFR of the quintiles to the probability of oCAD (P_oCAD_) using a power law:

$$\mathrm{P_{oCAD}}=a\cdot {x}^{-k},$$

where *x* is either globMFR or segMFR, and *a* and k are fit parameters.

### Follow-up

Patient follow-up was obtained by use of medical records. Our endpoint was the presence or absence of oCAD, as the purpose of Rb-82 PET is to assess the presence, extent, and functional importance of oCAD in order to tailor treatment. Patients were classified as having oCAD if they were clinically referred for invasive coronary angiography (ICA) during follow-up which was also classified as positive. It was to the discretion of the treating cardiologist whether a patient was sent for ICA or not, primarily based on a combination of (persistent) complaints, low MRF values, and high coronary calcium score. A positive ICA was defined by an intermediate or severe stenosis with a fractional flow reserve < 0.8 or > 70% stenosis in the left anterior descending (LAD), left circumflex (LCX) or right coronary artery (RCA), or > 50% stenosis in the left main coronary artery.

### Statistical analysis

Patient characteristics and continuous variables were expressed as mean ± standard deviation (SD) or median [interquartile range] as appropriate. Statistical analysis was performed using IBM SPSS (IBM SPSS Statistics for Windows, Version 26.0. Armonk, NY: IBM Corp). To assess differences between patient characteristics with visually normal and abnormal scans, the t test, Mann–Whitney *U* test, or *χ*^2^-test were performed. To determine goodness of fit between the probability function and our MFR data, we used *χ*^2^. The level of statistical significance was set to 0.05 for all statistical analyses.

## Results

Of all 1519 patients, 83% (1259) had a scan which was classified as normal and the remaining patients had a scan which was classified as abnormal. These two groups did not differ in weight, body mass index (BMI), and the risk factors such as smoking, hypertension, dyslipidaemia, diabetes, and family history (*P* ≥ .07), as shown in Table 1. Yet patients with abnormal scans were older, taller, and more often male (*P* ≤ .01). The median follow-up was 23 months [interquartile range: 18–27].

**Table 1:** Baseline characteristics of the patient population (*N* = 1519)

Characteristic	Visual normal (*N* = 1259)	Visual abnormal (*N* = 260)	*P* values
Age (years)	66 ± 11	69 ± 10	< .001
Male gender (%)	48	65	< .001
Weight (kg)	88 ± 20	90 ± 19	.07
Height (cm)	173 ± 10	175 ± 10	.01
BMI (kg·m^2^)	29 ± 6	30 ± 6	.47
Current smoking (%)	13	13	.99
Hypertension (%)	63	63	.98
Dyslipidemia (%)	42	45	.42
Diabetes (%)	20	23	.34
Family history (%)	52	48	.22
Segmental stress MBF	2.1 ± 0.6	1.4 ± 0.6	< .001
Segmental rest MBF	0.9 ± 0.3	0.8 ± 0.3	< .001
Segmental MFR	1.9 ± 0.5	1.4 ± 0.6	< .001
Global stress MBF	2.6 ± 0.7	2.0 ± 0.7	< .001
Global rest MBF	1.1 ± 0.4	1.1 ± 0.4	.024
Global MFR	2.5 ± 0.6	2.1 ± 0.7	< .001
Time to follow-up (months)	23 [18–28]	23 [18–26]	.18
ICA performed	7.9 (99)	50 (131)	< .001
Obstructive CAD (%)	3.7 (46)	39 (102)	< .001
Time to Obstructive CAD (months)	1.1 [0.7–1.8]	4.9 [1.4–11.3]	< .001
PCI during follow-up (%)	2.2 (28)	20 (52)	< .001
CABG during follow-up (%)	1.6 (20)	16 (41)	< .001
All-cause mortality (%)	2.7 (34)	5.8 (15)	.01

Data are presented as mean ± SD, median [interquartile range] or percentage

*ICA* invasive coronary angiography, *PCI* percutaneous coronary intervention, *CABG* coronary artery bypass graft

Of the 1259 patients with normal scans, 3.7% (46) had oCAD during follow-up. These 46 patients were older (66 vs. 71 years) and more often male (47 vs. 70%) than the other 1213 patients who had a visual normal PET. Of the 260 patients with visually abnormal scans, 39% (102) had oCAD during follow-up. Of these 260 patients, 136 patients had a small defect and 124 patients had a larger defect. The percentage of patients with oCAD was lower in the patients with a small defect (19%, 26/136) than in the patients with a larger defect (61%, 67/125 *P* < .001).

Looking at MBF and MFR, we found lower segMBFstress (2.1 vs 1.4), segMBFrest (0.8 vs 0.9), segMFR (1.4 vs. 1.8), and also lower globMBFstress (2.6 vs. 2.0), globMBFrest (1.1 vs. 1.0), and globMFR (2.0 vs. 2.4) in patients with visually abnormal scans than in patients with visually normal scans (*P* < .001), as shown in Table 1. Moreover, the same tendency in MBFstress and MFR decrease was observed comparing normal with small and small with larger defects (*P* < .001).

**Figure 1:**
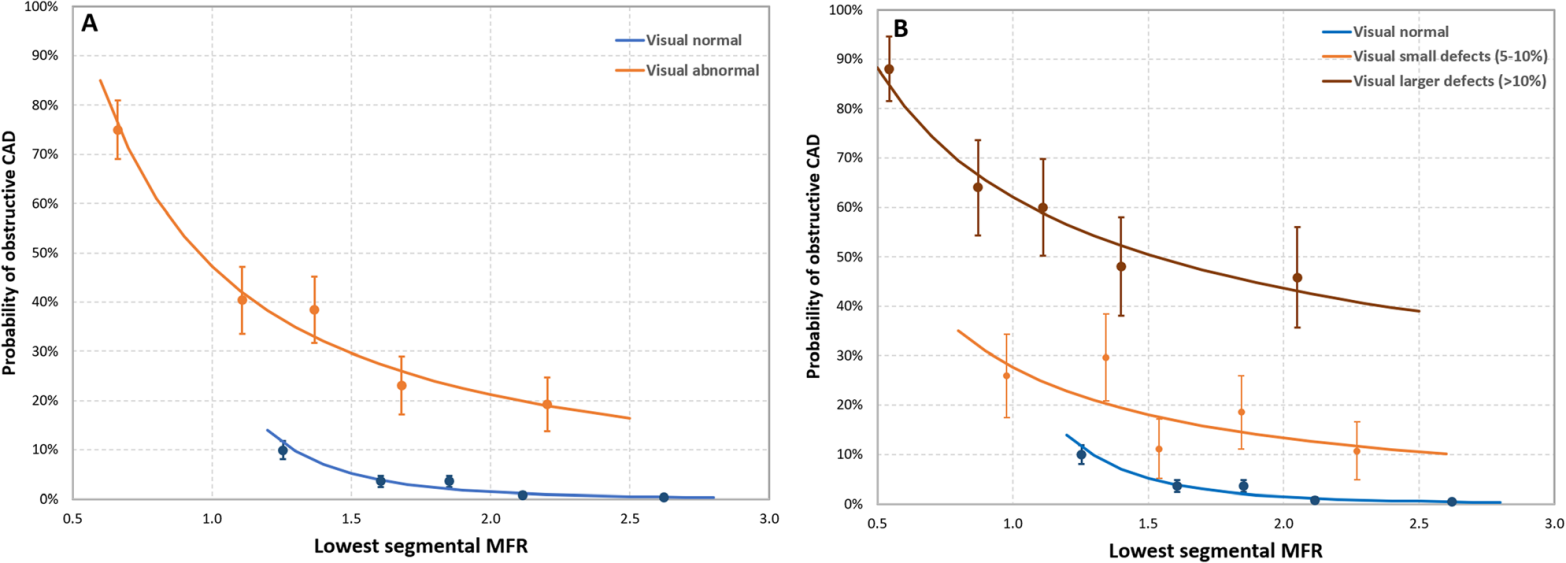
Plot of the mean of each quintile (dot with error bars) and the lines showing the patient’s probability (solid line) of having obstructive CAD for A) visually normal (blue) and abnormal (orange) scans and B) visually normal (blue), small defects (orange), and larger defects (brown) combined with the lowest measured segmental MFR. Each quintile contained 52 patients in the visual abnormal group, 27–28 patients in the small defect group, 24–25 patients in the larger defect group, and 251–252 patients in the visual normal group (Color figure online)

Combining *segmental* MFR with visual scan results, the patient’s probability of having oCAD increased with decreasing segMFR for both visually normal and abnormal scans, as well as for visually normal versus small and small versus larger defects, as shown in Fig. 1a and b. For the normal scans, the probability of oCAD increased from < 1% in patients with a segMFR ≥ 2.1% to 10% in patients with a segMFR of 1.3. For visually abnormal scans, the probability of oCAD increased from 10% in patients with a segMFR of 2.7% to > 70% for the patient group with a segMFR of 0.7. Moreover, the probability of oCAD increased from 10 to 40% in patients with a small defects versus 37% to 74% for patients with a larger defect for segMFR 0.7 to 2.7, respectively, as shown in Fig. 1b. The probability of oCAD can be described for visually normal scans by ${{P}_{\mathrm{oCAD}} =0.31 \cdot \mathrm{segMFR}}^{-4.47}$(*R*^2^ = 0.93), for visually abnormal scans by ${{P}_{\mathrm{oCAD}} =0.47\cdot \mathrm{segMFR}}^{-1.15}$ (*R*^2^ = 0.97), for small defects ${{P}_{\mathrm{oCAD}} =0.27\cdot \mathrm{segMFR}}^{-1.06}$ (*R*^2^ = 0.52), and larger defects ${{P}_{\mathrm{oCAD}} =0.98\cdot \mathrm{segMFR}}^{-0.42}$ (*R*^2^ = 0.84).

**Figure 2:**
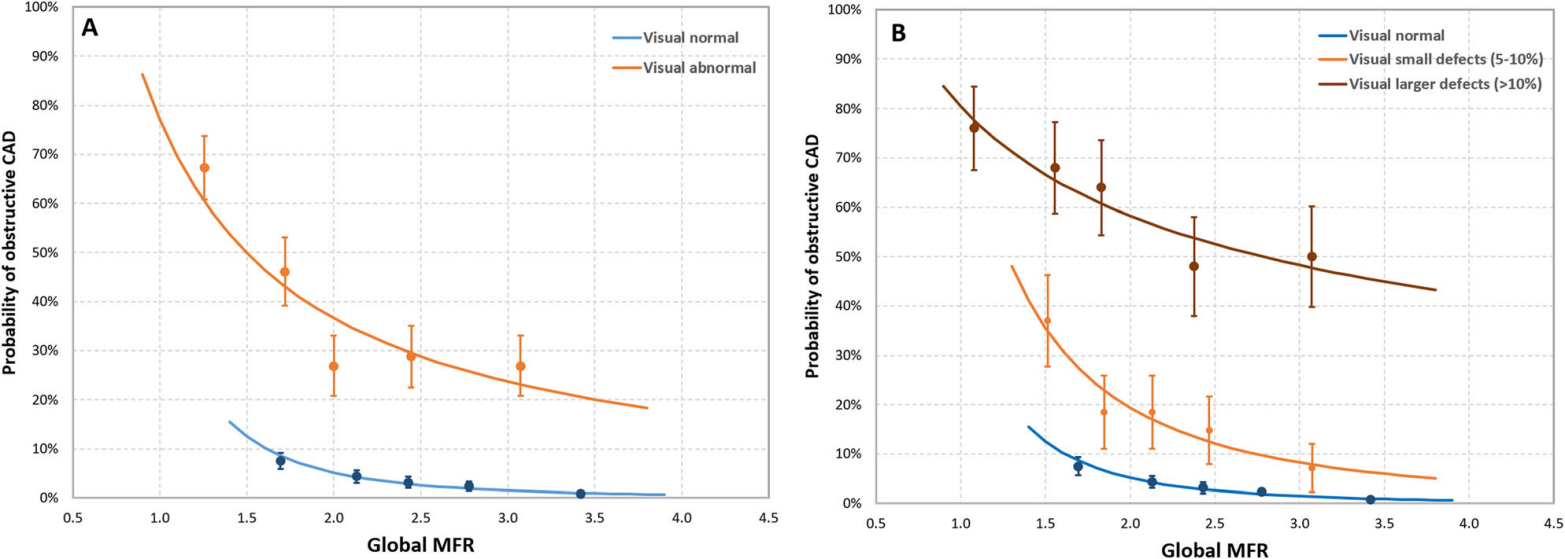
Plot of the mean of each quintile (dot with standard error bars) and the lines showing the patient’s probability (solid line) of having A) visually normal (blue) and abnormal (orange) scans and B) visually normal (blue), small defects (orange), and larger defects (brown) combined with the global MFR. Each quintile contained 52 patients in the visual abnormal group, 27–28 patients in the small defect group, 24–25 patients in the larger defect group, and 252 or 251 patients in the visual normal group (Color figure online)

When combining *global* MFR with visual assessment, we also observed an increase in the probability of oCAD with decreasing globMFR for both visually normal and abnormal Rb-82 PET scans, as shown in Fig. 2. For the normal scans, the probability of oCAD increased from 1% in patients with a globMFR of ≥ 3.4% to 13% in patients with a globMFR of 1.5. For visually abnormal scans, the probability of oCAD increased from 21% in patients with a globMFR of 3.4% to > 70% in patients with a globMFR of ≥ 1.1. Moreover, the probability of oCAD increased from 6% to 36% in patients with a small defects versus 46% to 76% for patients with a larger defect for globMFR 1.5 to 3.4, respectively, as shown in Fig. 2b

The probability of oCAD can be described for visually normal scans by ${P}_{\mathrm{oCAD}} =0.44\cdot {\mathrm{globMFR}}^{-3.07}$(*R*^2^ = 0.95), for visually abnormal scans by ${P}_{\mathrm{oCAD}} =0.77\cdot {\mathrm{globMFR}}^{-1.07}$ (*R*^2^ = 0.80), for small defects ${P}_{\mathrm{oCAD}} =0.83\cdot {\mathrm{globMFR}}^{-2.10}$ (*R*^2^ = 0.93), and larger defects ${P}_{\mathrm{oCAD}} =0.80\cdot {\mathrm{globMFR}}^{-0.47}$ (*R*^2^ = 0.90).

## Discussion

In this study, we estimated the patient’s probability of having oCAD based on the combination of visual assessment of Rb-82 PET scans and MFR values. Although a visual interpretation seems to be sufficient to discriminate patients with a probability > 10% from patients with a probability < 10% patients, our study showed that MFR can be used for a more patient-tailored risk assessment, as the probability of an individual patient having oCAD strongly depends on MFR (as shown in Figs. 1 and 2). Combining MFR measurements with visual interpretation of Rb-82 PET to estimate patient’s risk of having oCAD may, therefore, impact the treatment strategy.

Our results are in line with a previous study performed by Murthy et al who reported that the global MFR provides prognostic information in addition to only visual assessment.^6^ Murthy et al used cardiac mortality as primary endpoint, instead of oCAD as chosen in this study, and included a large number of patients with a prior history of CAD. They found that patients with a visually normal Rb-82 PET scan and low global MFR (< 1.5) had a higher annualized mortality rate (3.6%) as compared to patients with visually abnormal scans and high global MFR (1%). These results are in line with the ones presented in this study as the probability on oCAD for a normal interpreted scan with low globMFR or segMFR exceeded that of an abnormal scan with high globMFR or segMFR.

This study has several limitations. First, we only included patients without prior history of CAD. Therefore, the derived probability of a patient having oCAD might not be generalizable to patients with a prior history of CAD. However, as mentioned above, Murthy et al showed results that are in line with ours while including a large number of patients with a prior history of oCAD.^6^ We expect that the probability of oCAD also increases with decreasing (global or segmental) MFR and decreases with increasing (global or segmental MFR) for both visually normal and abnormal Rb-82 PET scans in patients with a prior history.

Second, it is possible that in some patients, the progression of CAD eventually resulted in obstructive CAD during the follow-up period of two years. These patients were, therefore, labeled as oCAD in this study while oCAD may not have been present at the time of the PET scan. However, only 3 out of the 102 patients diagnosed with oCAD in the visual abnormal group and 8 out of the 45 patients diagnosed with oCAD in the normal group were diagnosed after 12 months, limiting this effect.

Third, the probability of oCAD as a function of (global or segmental) MFR as derived in our study may not be similar for other centers. Both patient population and acquisition, reconstruction, and post-processing protocols may differ which can result in different MFR values^14–17^ and, hence, in a different probability function. However, we do not expect its shape to be different: patient’s probability to have oCAD is likely to depend strongly on MFR, for both visually normal and abnormal scans. Ideally, each center should derive its own relation between MFR and probability of oCAD.

Finally, the retrospective study design may have led to some bias in our study population, as only patients who were clinically indicated underwent ICA. We classified patients as having oCAD if follow-up included a conclusive ICA for oCAD. Inherently, we might have missed patients with oCAD as not all were referred for ICA, patients with small-moderate stenosis were not classified as having oCAD, and some of the deceased patients (*n* = 49) may have died from oCAD. This bias may have led to an underestimation of patients with oCAD and, consequently, to an underestimation of the probability of oCAD. This could partly explain the relatively low percentage of patient (39%) with an abnormal PET who were diagnosed with obstructive CAD. Yet previous studies show a comparable percentage of obstructive CAD after positive stress testing. Patel et al reported that that only 41% of the patients (*n* = 398.978) with a positive stress test did have oCAD (> 50% stenosis).^18^ These patients might suffer from functional abnormalities such as vasospastic angina or coronary microvascular disease (CMD) rather than obstructive CAD.^19^ However, we did not perform invasive coronary flow reserve or microvascular resistance measurements and could not confirm this. Nevertheless, we do not expect that the shape of the derived probability functions will change strongly due to this bias.

## New Knowledge Gained

In clinical practice, Rb-82 PET-based MFR in combination with visual assessment is used to diagnose oCAD as recently recommended by guidelines.^7,8^ However, in diagnosing oCAD, it is unclear how visual assessment can be combined best with MFR, especially when MFR is discrepant from the qualitative interpretation. We provided a probability function that can be used in clinical practice and is of particular value for patients with a normal PET scan and low MFR and vice versa. In these cases, the probability of a patient having oCAD is altered when compared to the probability when solely using visual or MFR assessment, possibly affecting treatment strategy. Hence, estimating patient’s risk on oCAD should be based on both MFR and visual interpretation of Rb-82 PET.

Ideally, other centers should recreate the probability function for their own settings as absolute values may differ from ours due to difference in patient population, PET scanner, acquisition, reconstruction, and post-processing techniques.^14–17^ However, we do expect to see a similar shape of this function with a strong dependence on MFR, for both visually normal and abnormal scans.

## Conclusion

In estimating the probability of a patient having oCAD using Rb-82 PET, both visual interpretation and MFR measurements are essential. Patients with a probability > 10% can be distinguished from patients with a probability < 10% based on visual interpretation only. However, there is a strong dependence of MFR on patient’s individual probability of having oCAD: these probabilities may range from < 1% to > 80%. Hence, combining both visual interpretation and MFR results in a better individual risk assessment which may impact treatment strategy.

### Supplementary Information

Below is the link to the electronic supplementary material.

Supplementary file1 (PPTX 913 kb)

## Abbreviations

oCAD: Obstructive Coronary artery disease
CABG: Coronary artery bypass grafting
ICA: Invasive coronary angiography
LAD: Left anterior descending
LCX: Left circumflex
LV: Left ventricle
MBF: Myocardial blood flow
MFR: Myocardial flow reserve
MPI: Myocardial perfusion imaging
PCI: Percutaneous coronary intervention
PET: Positron emission tomography
Rb-82: Rubidium-82
RCA: Right coronary artery
TAC: Time activity curve

## References

[1] Saraste A, Kajander S, Han C, Nesterov SV, Knuuti J (2012). PET: Is myocardial flow quantification a clinical reality?. J Nucl Cardiol.

[2] deKemp RA, Yoshinaga K, Beanlands RSB (2007). Will 3-dimensional PET-CT enable the routine quantification of myocardial blood flow?. J Nucl Cardiol.

[3] Sciagra R, Passeri A, Bucerius J, Verberne HJ, Slart RHJA, Lindner O (2016). Clinical use of quantitative cardiac perfusion PET: Rationale, modalities and possible indications: Position paper of the cardiovascular committee of the European association of nuclear medicine (EANM). Eur J Nucl Med Mol Imaging.

[4] Jaarsma C, Leiner T, Bekkers SC, Crijns HJ, Wildberger JE, Nagel E (2012). Diagnostic performance of noninvasive myocardial perfusion imaging using single-photon emission computed tomography, cardiac magnetic resonance, and positron emission tomography imaging for the detection of obstructive coronary artery disease a meta-analysis. JACC.

[5] Knuuti J, Ballo H, Juarez-Orozco LE, Saraste A, Kolh P, Rutjes AWS (2018). The performance of non-invasive tests to rule-in and rule-out significant coronary artery stenosis in patients with stable angina: A meta-analysis focused on post-test disease probability. Eur Heart J.

[6] Murthy VL, Naya M, Foster CR, Hainer J, Gaber M, Di Carli G (2011). Improved cardiac risk assessment with noninvasive measures of coronary flow reserve. Circulation.

[7] Blankstein R, Shaw LJ, Gulati M, Atalay MK, Bax J, Calnon DA (2022). Implications of the 2021 AHA/ACC/ASE/CHEST/SAEM/SCCT/SCMR chest pain guideline for cardiovascular imaging: A multisociety viewpoint. JACC.

[8] Murthy V, Bateman T, Beanlands R, Berman D, Borges-Neto S, Chareonthaitawee P (2018). Clinical quantification of myocardial blood flow using PET: Joint position paper of the SNMMI cardiovascular council and the ASNC. J Nucl Cardiol.

[9] Koenders SS, van Dijk JD, Jager PL, Ottervanger JP, Slump CH, van Dalen JA (2019). Impact of regadenoson-induced myocardial creep on dynamic rubidium-82 PET myocardial blood flow quantification. J Nucl Cardiol.

[10] Koenders SS, van Dijk JD, Jager PL, Ottervanger JP, Slump CH, van Dalen JA (2019). How to detect and correct myocardial creep in myocardial perfusion imaging using rubidium-82 PET?. J Nucl Cardiol.

[11] Dilsizian V, Bacharach SL, Beanlands RS, Bergmann SR, Delbeke D, Dorbala S (2016). ASNC imaging guidelines/SNMMI procedure standard for positron emission tomography (PET) nuclear cardiology procedures. J Nucl Cardiol.

[12] Lortie M, Beanlands R, Yoshinaga K, Klein R, DaSilva J, deKemp R (2007). Quantification of myocardial blood flow with 82Rb dynamic PET imaging. Eur J Nucl Med Mol Imaging.

[13] Koenders SS, van Dalen JA, Jager PL, Mouden M, Slump CH, van Dijk JD (2022). Diagnostic value of regional myocardial flow reserve measurements using rubidium-82 PET. Int J Cardiovasc Imaging.

[14] Koenders SS, van Dijk JD, Jager PL, Mouden M, Tegelaar AG, Slump CH (2021). Effect of temporal sampling protocols on myocardial blood flow measurements using rubidium-82 PET. J Nucl Cardiol.

[15] Murthy VL, Lee BC, Sitek A, Naya M, Moody J, Polavarapu V (2014). Comparison and prognostic validation of multiple methods of quantification of myocardial blood flow with 82Rb PET. J Nucl Med.

[16] Tahari AK, Lee A, Rajaram M, Fukushima K, Lodge MA, Lee BC (2014). Absolute myocardial flow quantification with (82)rb PET/CT: Comparison of different software packages and methods. Eur J Nucl Med Mol Imaging.

[17] Armstrong I, Tonge C, Arumugam P (2014). Impact of point spread function modeling and time-of-flight on myocardial blood flow and myocardial flow reserve measurements for rubidium-82 cardiac PET. J Nucl Cardiol.

[18] Patel MR, Peterson ED, Dai D, Brennan JM, Redberg RF, Anderson HV (2010). Low diagnostic yield of elective coronary angiography. N Engl J Med.

[19] Lee SH, Shin D, Lee JM, van de Hoef TP, Hong D, Choi KH (2022). Clinical relevance of ischemia with nonobstructive coronary arteries according to coronary microvascular dysfunction. J Am Heart Assoc.

