# Supplementary material for: Patient-tailored risk assessment of obstructive coronary artery disease using Rubidium-82 PET-based myocardial flow quantification with visual interpretation
Source: J Nucl Cardiol. 2023 Apr 19;30(5):1890–6. doi: 10.1007/s12350-023-03237-z (PMC10558363; doi:10.1007/s12350-023-03237-z)
Supplement: Supplementary file 1 — Supplementary file1 (PPTX 913 kb) [file 12350_2023_3237_MOESM1_ESM.pptx]

## Slide 1
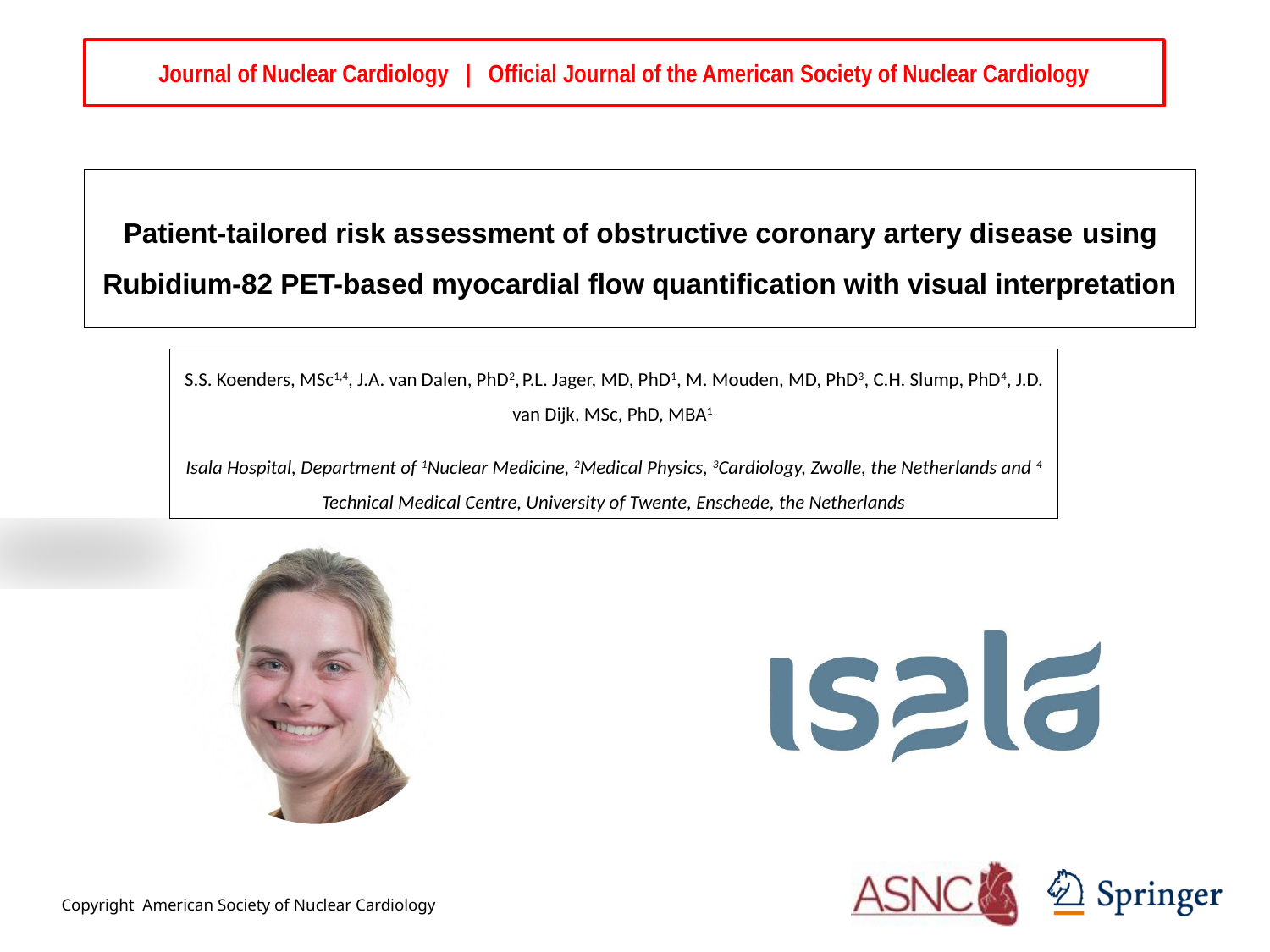

Journal of Nuclear Cardiology | Official Journal of the American Society of Nuclear Cardiology
# Patient-tailored risk assessment of obstructive coronary artery disease using Rubidium-82 PET-based myocardial flow quantification with visual interpretation
S.S. Koenders, MSc1,4, J.A. van Dalen, PhD2, P.L. Jager, MD, PhD1, M. Mouden, MD, PhD3, C.H. Slump, PhD4, J.D. van Dijk, MSc, PhD, MBA1
Isala Hospital, Department of 1Nuclear Medicine, 2Medical Physics, 3Cardiology, Zwolle, the Netherlands and 4 Technical Medical Centre, University of Twente, Enschede, the Netherlands
Copyright American Society of Nuclear Cardiology

## Slide 2
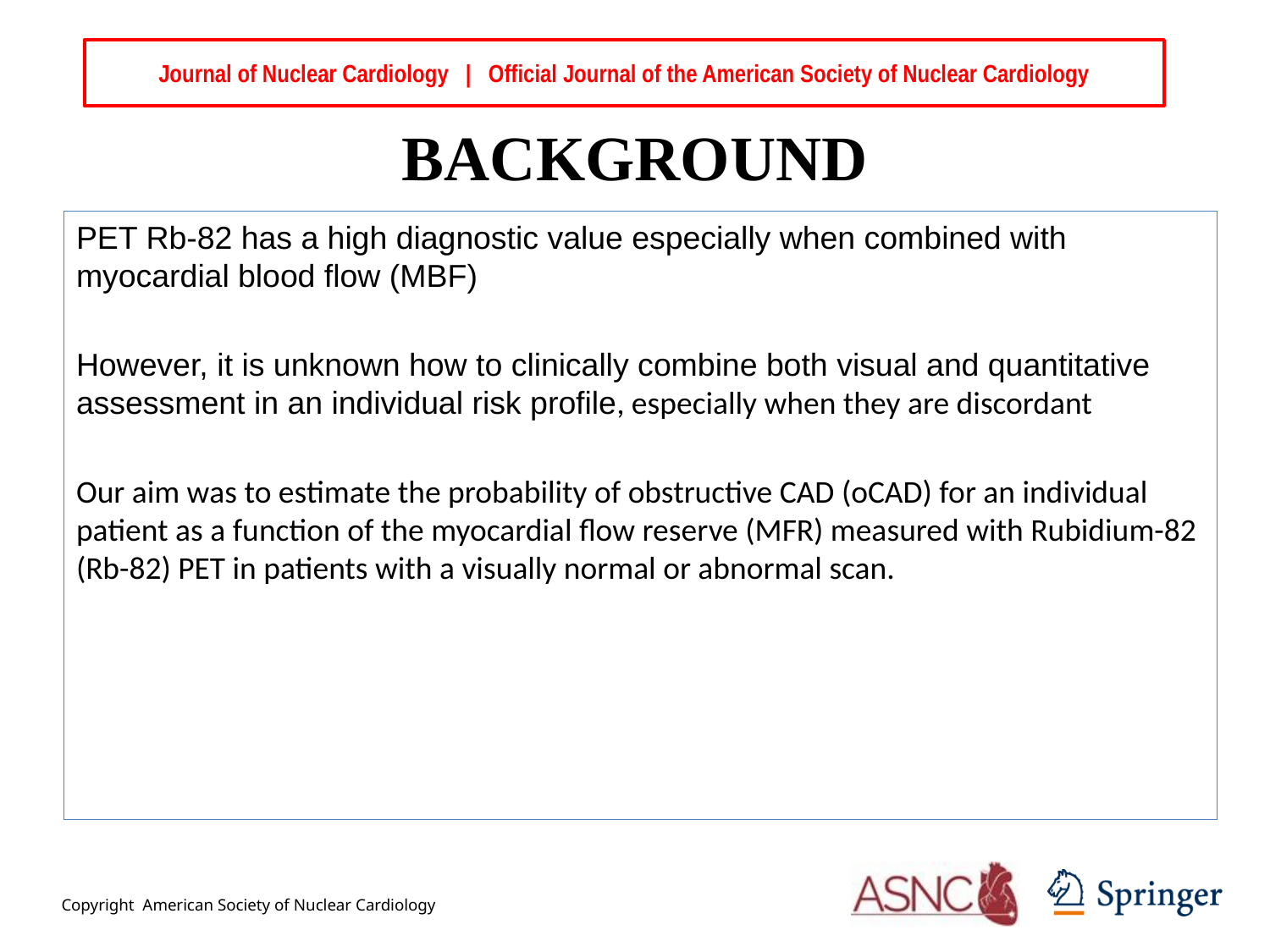

Journal of Nuclear Cardiology | Official Journal of the American Society of Nuclear Cardiology
# BACKGROUND
PET Rb-82 has a high diagnostic value especially when combined with myocardial blood flow (MBF)
However, it is unknown how to clinically combine both visual and quantitative assessment in an individual risk profile, especially when they are discordant
Our aim was to estimate the probability of obstructive CAD (oCAD) for an individual patient as a function of the myocardial flow reserve (MFR) measured with Rubidium-82 (Rb-82) PET in patients with a visually normal or abnormal scan.
Copyright American Society of Nuclear Cardiology

## Slide 3
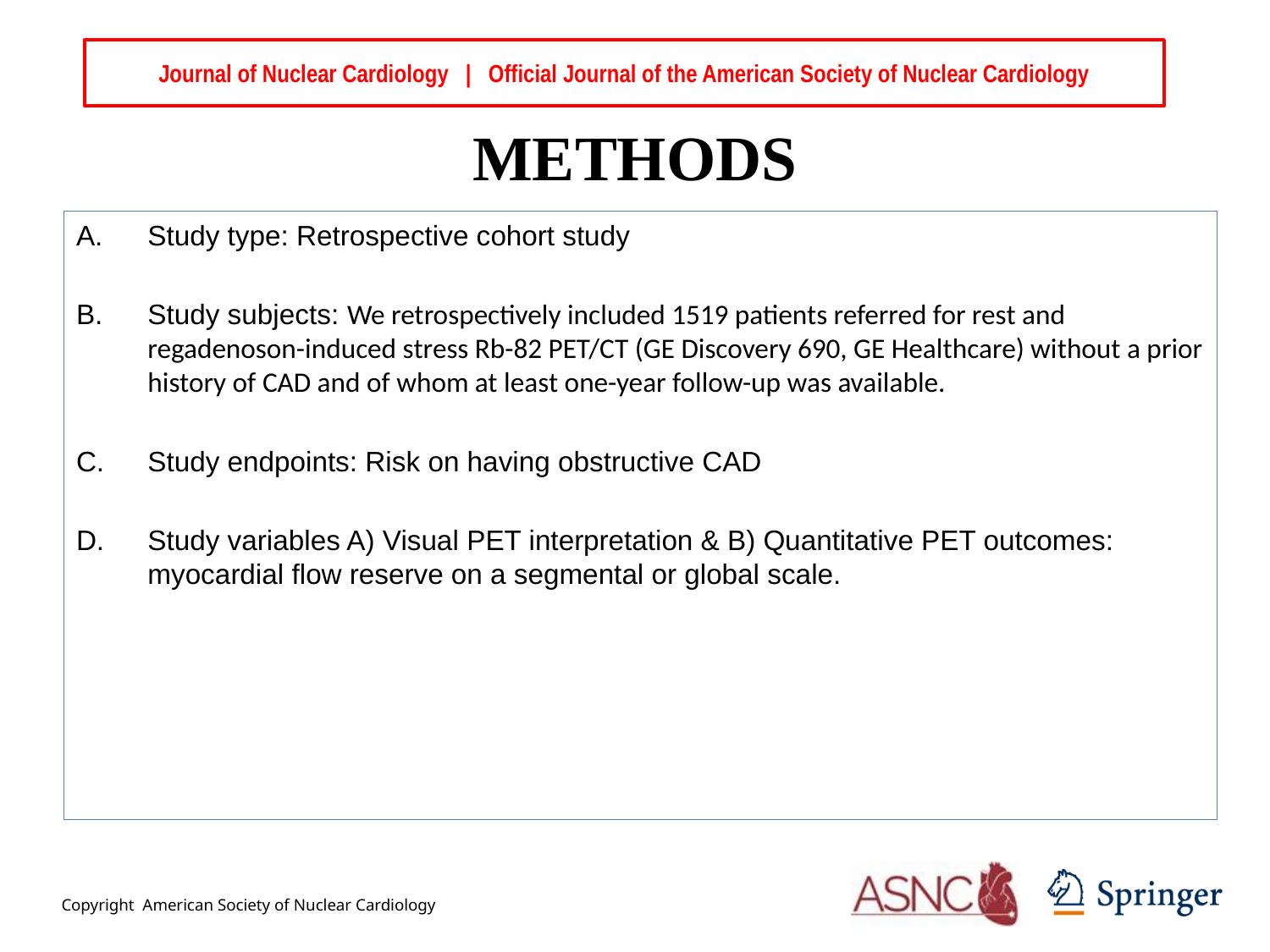

Journal of Nuclear Cardiology | Official Journal of the American Society of Nuclear Cardiology
# METHODS
Study type: Retrospective cohort study
Study subjects: We retrospectively included 1519 patients referred for rest and regadenoson-induced stress Rb-82 PET/CT (GE Discovery 690, GE Healthcare) without a prior history of CAD and of whom at least one-year follow-up was available.
Study endpoints: Risk on having obstructive CAD
Study variables A) Visual PET interpretation & B) Quantitative PET outcomes: myocardial flow reserve on a segmental or global scale.
Copyright American Society of Nuclear Cardiology

## Slide 4
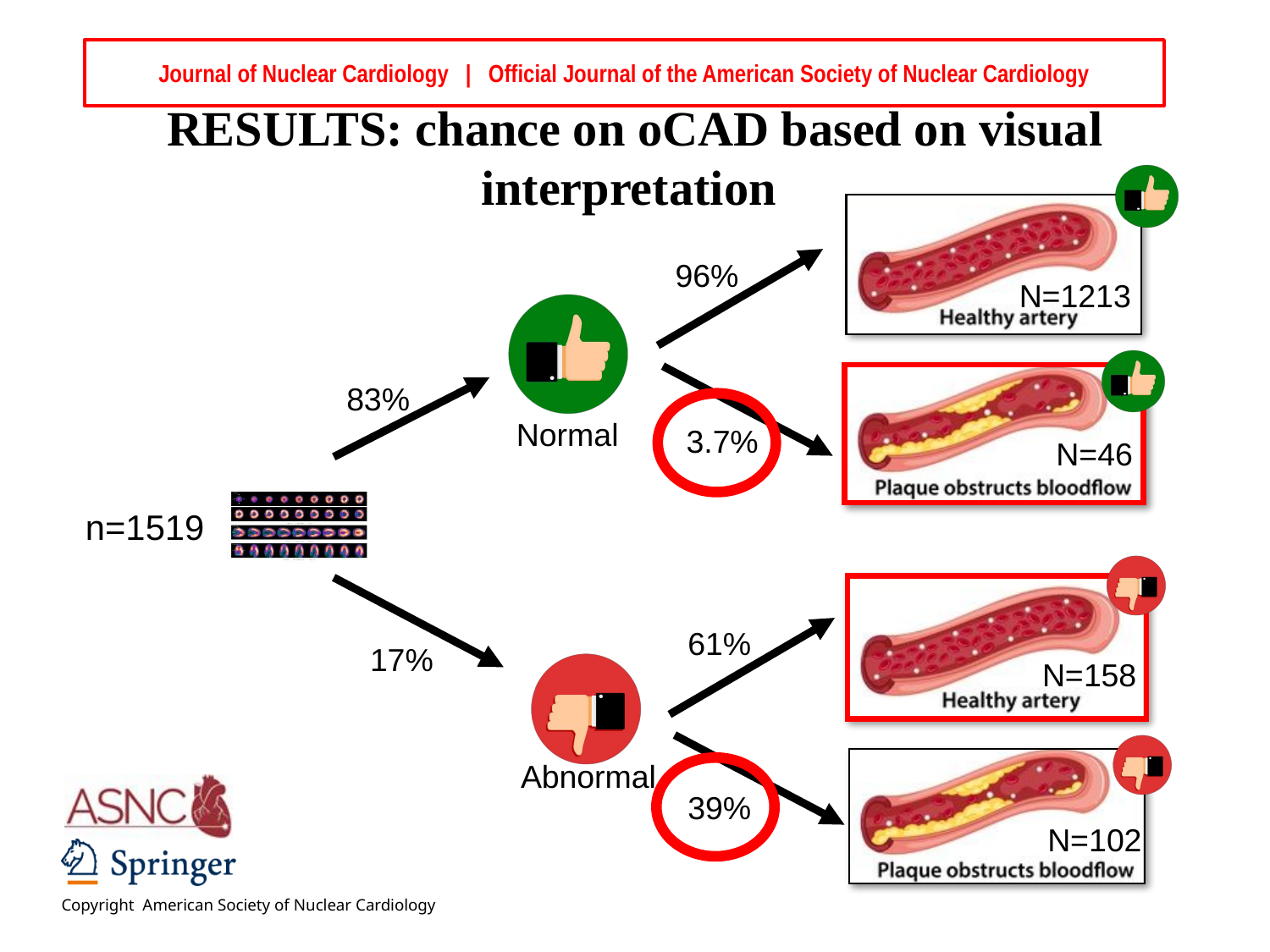

Journal of Nuclear Cardiology | Official Journal of the American Society of Nuclear Cardiology
# RESULTS: chance on oCAD based on visual interpretation
96%
N=1213
83%
Normal
3.7%
N=46
n=1519
61%
17%
N=158
Abnormal
39%
N=102
Copyright American Society of Nuclear Cardiology

## Slide 5
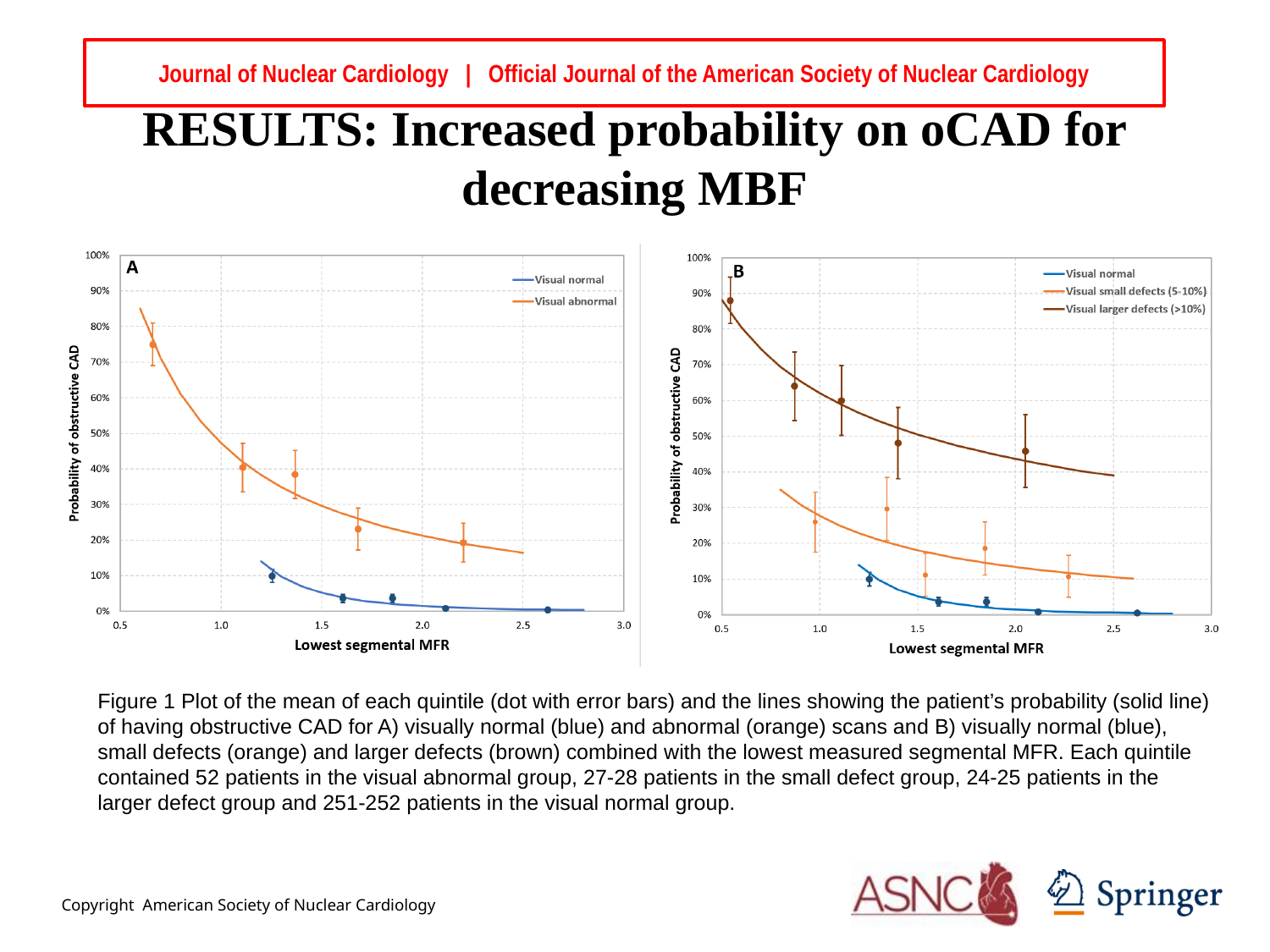

Journal of Nuclear Cardiology | Official Journal of the American Society of Nuclear Cardiology
# RESULTS: Increased probability on oCAD for decreasing MBF
Figure 1 Plot of the mean of each quintile (dot with error bars) and the lines showing the patient’s probability (solid line) of having obstructive CAD for A) visually normal (blue) and abnormal (orange) scans and B) visually normal (blue), small defects (orange) and larger defects (brown) combined with the lowest measured segmental MFR. Each quintile contained 52 patients in the visual abnormal group, 27-28 patients in the small defect group, 24-25 patients in the larger defect group and 251-252 patients in the visual normal group.
Copyright American Society of Nuclear Cardiology

## Slide 6
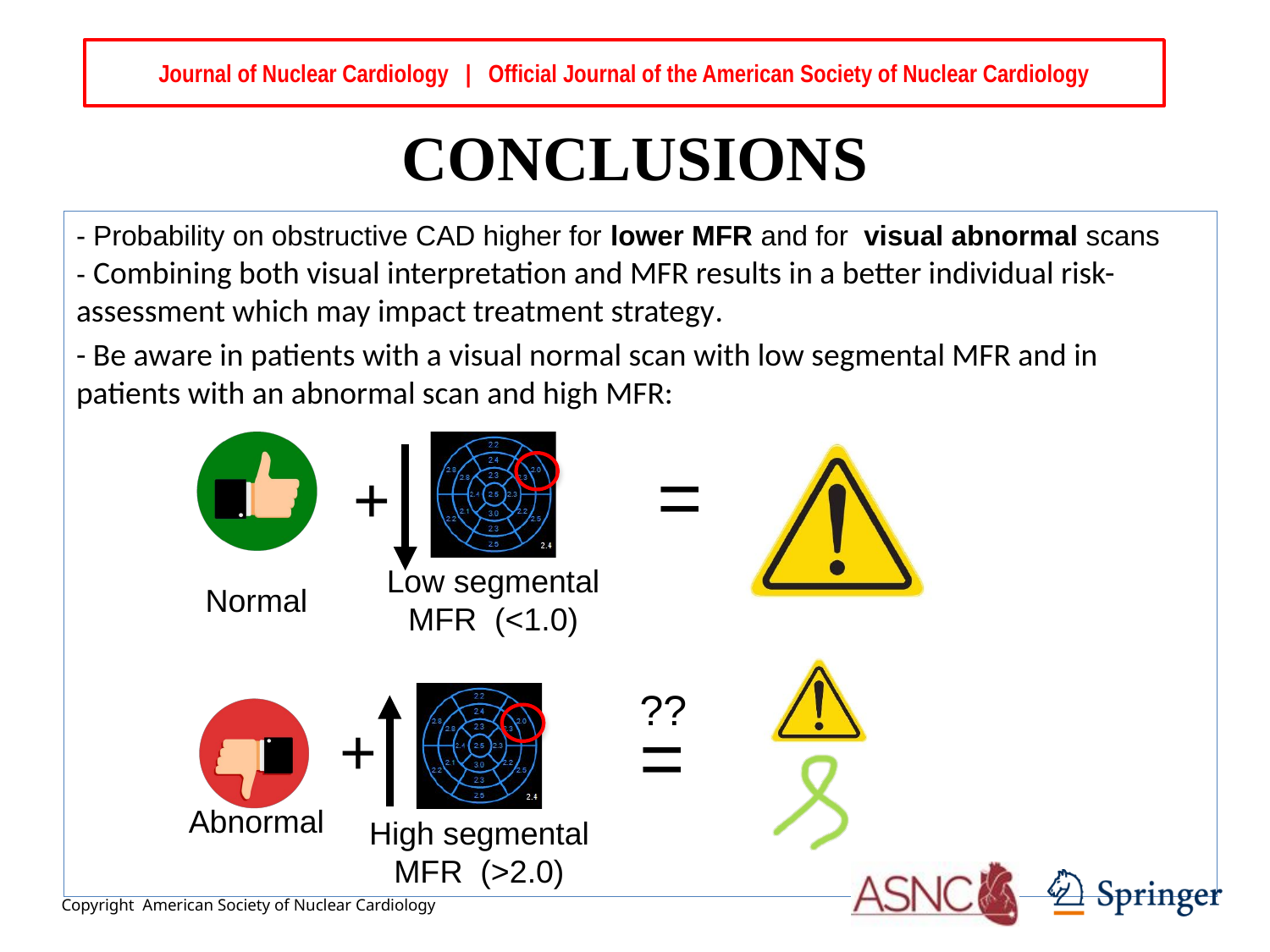

Journal of Nuclear Cardiology | Official Journal of the American Society of Nuclear Cardiology
# CONCLUSIONS
- Probability on obstructive CAD higher for lower MFR and for visual abnormal scans
- Combining both visual interpretation and MFR results in a better individual risk-assessment which may impact treatment strategy.
- Be aware in patients with a visual normal scan with low segmental MFR and in patients with an abnormal scan and high MFR:
=
+
Low segmental MFR (<1.0)
Normal
??
=
+
Abnormal
High segmental MFR (>2.0)
Copyright American Society of Nuclear Cardiology
